# Healthcare accessibility and financial risks for unmet health needs of the older adult population in the republic of Serbia

**DOI:** 10.3389/fpubh.2026.1912324

**Published:** 2026-09-01

**Authors:** Nikola Savić, Slobodanka Bogdanović Vasić, Branimirka Aranđelović, Katarina Pavić, Sanja Kocić, Aleksandra Arnaut, Milena Zlatanović, Jelena Stojčević-Maletić, Svetlana Ristić

**Affiliations:** 1 Singidunum University, Belgrade, Serbia; 2 Institute of Public Health of Serbia “Dr Milan Jovanović Batut”, Belgrade, Serbia; 3 University of Bijeljina, Faculty of Health Studies, Bijeljina, Bosnia and Herzegovina; 4 University of Novi Sad, Faculty of Medicine, Department of Nursing, Novi Sad, Serbia; 5 Academy of Vocational Studies Šabac, Šabac, Serbia; 6 University of Kragujevac, Faculty of Medical Sciences, Kragujevac, Serbia; 7 Academy of Vocational Studies Ćuprija, Ćuprija, Serbia; 8 University of Novi Sad, Faculty of Medicine, Department of Biochemistry, Novi Sad, Serbia; 9 University Clinical Center of Vojvodina, Center for Laboratory Diagnostics and Nuclear Medicine, Novi Sad, Serbia; 10 Institute of Oncology and Radiology of Serbia, Belgrade, Serbia

**Keywords:** older adult population, health services, national health survey, socioeconomic inequalities, unmet health needs

## Abstract

**Background and objectives:**

Unmet healthcare needs indicate gaps between healthcare services considered necessary and those actually received, reflecting the accessibility of the health system. This study aimed to examine the frequency of unmet healthcare needs and identify Andersen model predictors of unmet needs due to financial and other objective reasons among older adults in Serbia.

**Materials and methods:**

This cross-sectional study used data from the 2019 Serbian National Health Survey, conducted from October to December 2019 by the Institute of Public Health of Serbia “Dr Milan Jovanović Batut” and the Ministry of Health. The analysis included a representative sample of respondents aged 65 years and older.

**Results:**

Among 1,116 older adults with unmet healthcare needs, 40.5% reported financial reasons, while 59.5% reported other objective reasons. Most respondents had chronic health conditions, and 39.0% of them reported financial barriers to necessary healthcare (χ^2^ = 5.39, df = 1, *p* < 0.05). Respondents with poor self-rated health were more likely to report financial barriers than those with good self-rated health (OR = 1.492). Financial barriers were also more frequently reported by respondents who had visited their chosen physician (OR = 1.863), used emergency services (OR = 1.869), or used private healthcare services (OR = 2.069).

**Conclusion:**

Financial constraints represent an important barrier to healthcare access among older adults in Serbia. Designing more accessible healthcare models requires a transparent and comprehensive analysis of the economic position of older people and its impact on obtaining necessary healthcare services.

## 1 Introduction

Serbia belongs to the middle developed countries of Eastern Europe, where a little more than 6,500,000 inhabitants live. Demographically, Serbia is among the oldest populations in Europe, every fifth resident is 65 or older (1). The demographic old-age dependency ratio is defined as the number of individuals aged 65 and over per 100 people of working age defined as those aged between 20 and 64. The largest number of countries with prospective old-age dependency ratios (POADR) were in Europe. According to Eurostat, the old-age dependency ratio in European countries in 2022 was the highest in Italy, 37.5%, and in Serbia, 33.1%. Which puts Serbia in 9th place among European countries. According to POADR projections, the growth trend will continue in the future both in Europe and in Serbia, with a significant impact on the sustainability of healthcare financing (2). The analysis of the biological characteristics of the population serves to assess the priority health needs and is the basis for the distribution of resources and the planning of health care measures. The issue of financing, organization and implementation of health care is one of the most important social issues of every country. The Alma Ata Declaration called health as a human right and ensuring access to health care as a primary responsibility of governments. However, in the time of globalization, inequalities in healthcare are inevitable. Universal health coverage, which means that “people receive the health services they need without suffering financial hardship” represents a serious challenge even for the most developed countries of the world (3). For health policy makers, despite all the obstacles they encounter, this remains the primary goal, and the inability to achieve it shows the weaknesses of all current health care models and all the complexity of the problem (4). The reform of the healthcare system in Serbia began in the 90s of the last century, with the collapse of the country and the civil war. The health system, which is a mixture of Soviet Semashko and German Bismarck models, underwent major structural and financial changes aimed at creating a sustainable health system (5).

Today, health care services for older people depend much more on economic resources and less on sociodemographic and cultural factors. There are two types of health insurance in Serbia: mandatory and voluntary. The state, through the Republic Health Insurance Fund, plays a key role in the social health insurance system. More than 97% of the population has compulsory health insurance. The costs of compulsory health insurance are borne by the insured by paying contributions on the realized income. In Serbia, almost 3/4 of the total current health expenditure are financed by public sources, mostly through the Republic Health Insurance Fund. Although the system is comprehensive and universal, with free access to public health services and defined by the Law on Health Insurance, there are large inequalities in access to health care (6).

According to the Andersen healthcare utilization model, there are three factors that contribute to the use of health services: predisposing factors, enabling factors and need factors. The predisposing factors represent the sociodemographic characteristics of individuals. The enabling factors represent factors that directly or indirectly enable the realization of the necessary health care, and they relate primarily to the possession of health insurance and the financial condition of the individual. The need factors imply the individual’s ability to adequately assess his own health and the need for health services, or he needs to be pointed out by a health professional (7).

The definition of the quality of health care, from Avesis Donabedian and “Evaluating the Quality of Medical Care” to today, has been constantly changing, so the former center has moved from the provider of health services to the users (8). Today, the quality of health care is considered to represent the degree to which health services increase the likelihood of desired health outcomes for the individual, in accordance with current professional knowledge. Quality health care is effective, efficient, accessible, timely, and safe, which leads to the improvement of the health of its citizens. Indicators of the quality of health care, subjective and objective, enable the comparison of several health care systems, as well as the monitoring, management and creation of health policy within individual health care systems. Waiting lists, the distance of health institutions and financial limitations, as objective indicators, are most often used to assess the possibility of achieving the necessary health care and the presence of unmet health needs, as well as the analysis of health system reforms (9).

The aim of research was to determine the frequency and predictive influence of factors from the Andersen healthcare utilization model on the impossibility of achieving the necessary health care from financial and other objective factors among the older adults (65 and more years) in Serbia.

## 2 Materials and methods

### 2.1 The data report methods

National Health survey Serbia 2019 Database was the last National Health survey Serbia conducted in 2019. The research was conducted as a cross-sectional study, and the sample was random, stratified two-phase sample without repetition, realized according to the instruments and methodology of the European Health Interview Survey wave 2 (10).

The basic sample consisted of all households listed in the census. The method of stratification and multiple-phase sampling was used to obtain a random research sample. There were a 4 main stratum in the sample: Vojvodina, Belgrade, Šumadija and Western Serbia, South-East Serbia. Each stratum was divided into cities and other (rural) regions. The sample formed in this way enables a comparative analysis of (un)achieved care quality among the oldest residents (65 and more years) both at the national level and between strata and between cities and rural areas.

Two-phase sampling includes local communities, selected on the basis of probability proportional to their size and households as units of the second phase selected on the basis of linear sampling method with the random start and uniform selection steps. The assessment of multicollinearity among independent variables did not indicate the presence of significant multicollinearity in the analyzed model.

The analysis does not include residents of Kosovo, nor persons staying in specialized institutions, homes for the older adults, psychiatric institutions, prisons.

### 2.2 Survey data description

Out of a total of 15,621 respondents from 5,114 households, 3,540 of them were aged 65 and more years. For the purposes of the research, respondents who had a need for health care, but did not realize it, were selected, *n* = 1,116. Depending on the reason for not obtaining health care, the respondents were divided into two groups:

- Unmet health care needs due to financial reasons, *n* = 452.
- Unmet health care needs due to other reasons, *n* = 664.

Other reasons were the distance of the health facility and the waiting list, and both reasons.

### 2.3 Research instruments

Two types of questionnaires have been used in the research: the questionnaires about the household for information on the characteristics of the household and the face to face interview which provided data on health care, demographic and socio-economic characteristics of the interviewees. Each interviewee was familiar with the type and purpose of the research and each of them gave their written consent. The National Health Survey 2019 was carried out by the Ministry of Health of the Republic of Serbia. Ethical standards in the research were in accordance with the international ethical standards as well as with the Serbian legislation. Survey weights are included in all conducted analyses, and the entire procedure of statistical data processing was carried out in accordance with the methodology of the European Population Health Survey (EHIS).

### 2.4 Variables

The dependent variable was unmet needs for health care. The unmet needs for health care was measured as a binary response, only financial and other reasons. The independent variables according to the Anderson model were:

- The predisposing factors: age, gender, place of residence, marital and work status, knowledge of healthcare system.
- The enabling factors: financial condition of the user.
- The need factors: self-assessment of health, presence of chronic disease, use of health care services: visit to the chosen doctor, hospital treatment, emergency medical assistance service, services of a private doctor, screening examination, satisfaction with the health service.

This scientific study examines the financial reasons for the unmet health needs of the older adult population, as they have been identified as the main reason for the unmet needs of the population of the Republic of Serbia.

### 2.5 Statistical analysis

This study used univariate analysis to examine the characteristics of the sample. The binary logistic regression was used to examine the predicating factors of unmet needs for health care for financial and other reasons. First, variables were tested with a univariate model, and then statistically significant predictors were included in a multivariate regression model. The predicted value was described using adjusted odds ratios at 95% confidence intervals. In this study, univariate analysis was used as an initial step in identifying variables potentially associated with the outcome under study, after which statistically significant variables were included in the multivariate model.

The statistical analysis was performed using a software package, the Statistical Package for Social Sciences (SPSS) software version 19.0 (SPSS Inc., version 19.0, Chicago, IL, United States). The results were presented for a significance level of *p* ≤ 0.05.

## 3 Results

The survey included 676 (60.6%) women and 440 (39.4%) men. The average age of the subjects was 73.6 ± 6.1 years (range 65–99 years). Out of a total of 1,116 respondents, 40.5% of them did not obtain the necessary health care for financial reasons only, and 59.5% of them for other reasons. Table 1 shows the differences between the two tested groups of respondents in terms of their sociodemographic characteristics and health characteristics. Every third man and 42.9% of women cite a lack of finances as a reason for missing the necessary health care (χ^2^ = 4.09, df = 1, *r* < 0.05). As the level of education increases, the percentage of respondents who lacked health care due to financial limitations decreases (χ^2^ = 6.25, df = 2, *r* < 0.05). Similarly, the wellbeing index shows that, in contrast to the poor, the rich most often complain about the lack of health care for other reasons (χ^2^ = 15.82, df = 2, *r* < 0.01). The distribution of the reasons for missing health care among respondents who assess their own health as good and/or average is similar. Every third patient who is in poor health cites finances as the main reason for missing the necessary health service (χ^2^ = 9.38, df = 2, *r* < 0.05). The largest number of respondents have a chronic health disorder and 39% of them fail to realize health care due to financial limitations (χ^2^ = 5.39, df = 1, *r* < 0.05) The Wellbeing Index in the Serbian Population Health Survey is a composite measure of the material and socioeconomic status of a household, based on data on housing conditions and possession of various goods, rather than directly on income. Individual indicators are weighted using factor analysis, after which an overall wellbeing score is calculated for each household. Based on the score obtained, respondents are classified into five quintiles, from the poorest to the richest segment of the population (Table 1).

**Table 1:** The predisposing and the enabling factors in relation to reason for unmet health needs.

Variables	Categories	Unmet health needs for		*p*
		Only financial reasons *n* (%)	Other reasons *n* (%)	
Sex	Male	162 (36.8)	278 (63.2)	<0.05*
	Female	290 (42.9)	386 (57.1)	
Age	65–69	149 (43.3)	195 (56.7)	>0.05
	70–74	118 (41.5)	166 (58.5)	
	75–79	115 (39.7)	175 (60.3)	
	80 and more	70 (35.4)	128 (64.6)	
Education	Without finishing school	288 (43)	381 (57)	<0.05*
	High School	119 (38.8)	188 (61.2)	
	College/university	45 (32.1)	95 (67.9)	
Marital status	Married	242 (39.6)	369 (60.4)	>0.05
	Other	210 (41.6)	295 (58.4)	
Type of settlement	City	243 (40.5)	357 (59.5)	>0.05
	Other	209 (40.5)	307 (59.5)	
Region	Vojvodina	145 (44.5)	181 (55.5)	>0.05
	Belgrade	103 (34.8)	193 (65.2)	
	Sumadia/Western Serbia	98 (42.6)	132 (57.4)	
	Southern/Eastern Serbia	106 (40.2)	158 (59.8)	
Wellbeing index	The poorest	295 (43.8)	379 (56.2)	<0.01*
	Middle layer	80 (43.4)	104 (56.6)	
	The richest layer	77 (29.8)	181 (70.2)	
Self-assessment of health	Good	57 (43.8)	73 (56.2)	<0.05*
	Average	176 (45.8)	208 (54.2)	
	Poor	219 (36.4)	383 (63.6)	
Chronic disease	Yes	379 (39)	593 (61)	<0.05*
	No	69 (49.3)	71 (50.7)	

χ^2^ test. *Statistical significance.

In the previous year, 9 out of 10 respondents used primary health care services. Among users of primary health care services, 38.7% did not realize the necessary health service due to financial limitations (χ^2^ = 13.45, df = 1, *r* < 0.05). Every fourth respondent who used the services of Emergency Medical Assistance in the previous 12 months had financial difficulties due to which he did not receive the necessary health care (χ^2^ = 16.01, df = 1, *r* < 0.05). The oldest residents of Serbia most often do not use the services of private practice, and those who do most often do not have a financial, but other reason for unmet needs for healthcare (χ^2^ = 25.49, df = 1, *r* < 0.05). According to the wellbeing index, the services of a private doctor are most often used by the richest, and least often by the poorest (χ^2^ = 101.28, df = 2, *r* < 0.01). As satisfaction with the work of health services increases, the percentage of respondents who have unmet health needs for other reasons decreases (χ^2^ = 9.38, df = 2, *r* < 0.01). Most of the respondents have never done screening tests. Among women, the financial reason for unmet health needs was given by every second woman who had never had a Pap test and every third woman who had had a Pap test more than a year ago (χ^2^ = 7.82, df = 2, *r* < 0.05) (Table 2).

**Table 2:** The need factors in relation to used health care.

Variables	Categories	Unmet health needs for		*p*
		Financial reasons *n* (%)	Other *n* (%)	
A visit to the chosen doctor	Yes	390 (38.7)	617 (61.3)	<0.01*
	No	62 (56.9)	47 (43.1)	
Hospital treatment	Yes	68 (34.3)	130 (65.7)	>0.05
	No	384 (41.8)	534 (58.2)	
Emergency assistance services	Yes	48 (27)	130 (73)	<0.01*
	No	404 (43.1)	534 (56.9)	
Services of a private doctor	Yes	52 (25)	156 (75)	<0.01*
	No	400 (44.1)	508 (55.9)	
Satisfaction with the health service	Dissatisfied	62 (22.8)	210 (77.2)	<0.01*
	Neither satisfied, nor displeased	103 (37.6)	171 (62.4)	
	Satisfied	276 (50)	276 (50)	
Test for occult bleeding in the stool	Previous 12 months	19 (38)	31 (62)	>0.05
	More than 12 months	40 (38.1)	65 (61.9)	
	Never	381 (40.5)	560 (59.5)	
Mammography	Previous 12 months	16 (48.5)	17 (51.5)	>0.05
	5 and more years ago	59 (39.1)	92 (60.9)	
	Never	210 (44.2)	265 (55.8)	
PAP test	Previous 12 months	20 (44.4)	25 (55.6)	<0.05*
	More than 12 months	100 (36.8)	172 (63.2)	
	Never	155 (48.1)	167 (51.9)	

χ^2^ test. *Statistical significance.

Tables 3, 4 show the predictive significance of the socio-demographic characteristics, health status and use of healthcare of the respondents as regards the reason for unmet health needs. Male respondents cite financial reasons for not obtaining health care 1.3 times more often, respondents with secondary education 1.2 times, and those with higher education 1.6 times more often compared to respondents without education. The rich almost twice as often (1.8×) cite finances as a reason for not getting the necessary health care compared to the poor. Respondents who are in poor health are 1.3 times more likely to not receive health care for financial reasons compared to respondents who are in good health, and respondents who have a chronic disease are 1.5 times more likely. The wellbeing index made the most significant contribution to the multivariate regression model, the richest respondents mentioned money for unmet health needs 2× more often (OR = 1.919) compared to the poorest. At the same time, patients who are in poor health, according to their own assessment, almost 1.5 times more often (OR = 1.492) cite a lack of finances as the main reason for not obtaining health care compared to respondents who are in good health (Table 3).

**Table 3:** Regression analysis of the influence of the predisposing and the enabling factors on the reason for unmet health needs.

Variables (refrent value)	Univariate model		Multivariate model	
	ОR (95% CI)	*p*	ОR (95% CI)	*p*
Sex (Female)				
Male	1.289 (1.008–1.65)	<0,05*	1.298 (0.993–1.696)	>0.05
Age (65–69 years)				
70–74 years	1.075 (0.782–1.477)	>0.05	/	
75–79 years	1.163 (0.846–1.597)			
80 and more years	1.397 (0.974–2.005)			
Education (No school)				
High School	1.194 (0.906–1.574)	<0,05*	1.02 (0.748–1.39)	>0.05
College/university	1.596 (1.084–2.348)		1.238 (0.791–1.937)	
Type of settlement (Urban)				
Rural	1.000 (0.787–1.271)	>0.05	/	
Region (Vojvodina)				
Belgrade	1.501 (1.086–2.075)	>0.05	/	
Sumadia and Western Serbia	1.079 (0.768–1.517)			
Southern and Eastern Serbia	1.194 (0.859–1.659)			
Wellbeing index (The poorest)				
Middle layer	1.012 (0.728–1.406)	<0,01*	1.015 (0.718–1.437)	<0.05*
The richest layer	1.83 (1.346–2.487)		1.919 (1.355–2.717)	
Self-assessment of health (Good)				
Average	0.923 (0.618–1.377)	<0,05*	0.962 (0.635–1.458)	<0.05*
Poor	1.366 (0.93–2.005)		1.492 (0.98–2.272)	
Chronic disease (No)				
Yes	1.521 (1.066–2.17)	<0.05*	1.397 (0.949–2.057)	>0.05

OR, odds ratio; CI, confidence interval. *Statistical significance.

**Table 4:** Regression analysis of the influence of the need factors on the reason for unmet health needs.

Variables (refrent value)	Univariate model		Multivariate model	
	ОR (95% CI)	*p*	ОR (95% CI)	*p*
Primary health care services (No)				
Yes	2.087 (1.399–3.112)	<0.01*	1.863 (1.047–3.313)	<0.05*
Hospital treatment (No)				
Yes	1.375 (0.997–1.896)	0.05*	>0.05	
Emergency assistance services (No)				
Yes	2.049 (1.436–2.923)	<0.01*	1.869 (1.165–2.999)	<0.05*
Services of a private doctor (No)				
Yes	2.362 (1.681–3.319)	<0.01*	2.069 (1.279–3.347)	<0.05*
Satisfaction with the health service (satisfied)				
Neither satisfied, nor displeased	1.66 (1.235–2.232)	<0.01*	1.632 (1.098–2.424)	<0.01*
Dissatisfied	3.387 (2.438–4.706)		2.738 (1.762–4.256)	
Test na okulno krvarenje u stolici (never)				
Previous 12 months	1.11 (0.618–1.994)	>0.05	/	
5 and more years ago	1.106 (0.73–1.674)			
Mammography (previous 12 months)				
5 and more years ago	1.468 (0.688–3.128)	>0.05	/	
Never	1.188 (0.586–2.407)			
PAP test (never)				
Previous 12 months	1.16 (0.62–2.173)	<0.05*	0.999 (0.51--1.956)	>0.05
5 and more years ago	1.596 (1.148–2.219)		1.397 (0.986–1.98)	

OR, odds ratio; CI, confidence interval. *Statistical significance.

Respondents who in the previous 12 months used primary health care services, emergency medical assistance services, private doctor services, who were treated in a hospital and who were dissatisfied with the organization and work of the health service, as well as women who had a PAPA test, often cited financial reasons for not providing the necessary health care.

Financial limitations as the main reason why they missed the necessary health service were cited 1.8 times more often by respondents who visited their chosen doctor (OR = 1.863) in the previous year, as well as by respondents who used emergency services (OR = 1.869), 2 times more often (OR = 2,069) respondents who used the services of a private doctor. The strongest predictor was satisfaction with the health service, respondents who are dissatisfied with the work of the health service almost 3 times more often (OR = 2.738), and respondents who are neither satisfied nor dissatisfied 1.6 times more often (OR = 1.632) cite financial restrictions as an obstacle in the realization of the necessary of health care in comparison with respondents who are satisfied with the work of the health service (Table 4).

## 4 Discussion

Silver tsunami is an expression that is used more and more often to describe population aging and all the changes that such a change brings, especially in the domain of economy and health (11). In Serbia, only in the period between the two National Health Surveys, the share of old residents (65 and more years) increased by almost 5%, from 17.4% in 2013 to 22.1% in 2019. Consequently, the average age also increased from 42.2 to 43.9 years (11, 12).

The Gross Domestic Product (GDP) in Serbia was 51.51 billion US dollars in 2019 (13) According to the World Health Organization Global Health Expenditure current health expenditure was 8.67% of GDP in 2019 (14). Although the percentage of GDP allocated to health is relatively high, due to the low GDP the absolute allocation is significantly lower compared to the countries of the European Union (1,677.06 USD in Serbia compared to 3,493.6 USD in the EU in 2019 years) (15).

Despite the fact that in Serbia, almost the entire population aged 65 and over is covered by compulsory health insurance, the results of our research indicate that compulsory health insurance alone is often not enough to provide the oldest citizens with the necessary health care. According to the data of the last National health survey in Serbia, every third resident aged 65 and over had difficulties in realizing health care despite compulsory health insurance. Allocation for health care in relation to total spending is less than 60%, which ranks Serbia, along with some Eastern European countries, among the European countries that allocate the least for health care. The rest (slightly more than 40%) represents private allocation for healthcare (16). This coincides with the results of our research, in which 40.5% of respondents did not receive the necessary health care due to financial reasons. The analysis of private allocations for healthcare indicates that the largest part is made up of “out-of-pocket” direct payments. The experience of “out-of-pocket” spending varied across countries, in Serbia this percentage is almost twice as high as in EU countries (17).

It is a worrying fact that respondents who need health care the most, those who assess their own health as poor, as well as the chronically ill, fail to obtain the necessary health care for financial reasons. Possible causes of this are the lack of awareness of the importance of controlling and treating chronic diseases in the older adults, as well as social inequality, which is often felt by the poorest (18). According to some studies, the problem is in implementation and the establishment of a quality health care system for the older adults with multimorbidities is the existence of a “single-disease” model of health care which emphasizes acute/episodic care for one condition at a time (19, 20).

In addition to financial limitations, the uneven distribution of healthcare workers across the country, the lack of certain specialties and especially the departure of doctors and nurses to more developed EU countries make it even more difficult to achieve the necessary healthcare (21). In Serbia, there is a system of selected doctor in primary health care. Research shows that the general practitioner occupies a central place in providing health care to the older adult, and chronic health disorders and multimorbidities are the most common conditions that the chosen doctor encounters in primary health care (22). Current actions of the Ministry of Health with the Institute for Public Health are aimed at strengthening the capacity of primary health care and improving access to preventive health services with a special emphasis on screening programs. Similar to our findings, there are numerous other studies in which it was confirmed that out-of-pocket payments and co-payments are the main limiting factors for achieving the necessary health care among the oldest who consulted a chosen doctor (23–25).

In our research, the place of residence in terms of the distance to the health facility had no influence on the frequency of use of Emergency Medical Services. However, research conducted in Korea shows that the distance to the health facility, along with the health status of the respondents, is the main predictor of the use of Emergency Medical Services among older adult Koreans (26). Patient satisfaction with health care is an important predictor between expected and realized health services. Basically, satisfaction is a subjective experience of the patient, which is impossible to measure, but which is strongly related to the quality of the healthcare system (27, 28). The results of our research indicate that respondents who had financial difficulties in realizing the necessary health care are the least satisfied with the work of health services. In recent years, developed countries have invested huge amounts of money in improving the quality of health care and consequently increased patient satisfaction. The European countries that are most successful in this are Sweden, Germany and Norway (29). Research conducted in Spain shows a significant connection between investment in the healthcare system and patient satisfaction, in which spending on the healthcare system was 9.3% of GDP in 2019 and an increase of 5% over 2018. Increased funds were invested in order to reduce the lists waiting for medical treatment and increased satisfaction of users of health services (30). Similarly, the analysis of satisfaction in Indian hospitals has identified profiteering attitude as the reason for the patient’s satisfaction with the health service, which is correlated with the low rate of unmet health needs (31).

### 4.1 Study limitations

This study has several limitations that should be considered when interpreting the results. First, the cross-sectional design of the study allows for the identification of associations between the factors examined and unmet health needs, but does not allow for the determination of time series or conclusions about cause-and-effect relationships. The data are largely based on self-report, which cannot be ruled out as a possibility of recall bias, especially regarding previous use of health services and reasons for not using them. Although a wide range of sociodemographic, economic, and health characteristics were included in the analysis, the possibility of residual confounding due to unmeasured or unavailable factors remains. In addition, the study did not include institutionalized older adults, including those in nursing homes and other specialized facilities, or residents of Kosovo. This limitation is particularly important given that institutionalized older adults may have greater health needs and different patterns of health care utilization. Finally, the data were collected before the COVID-19 pandemic, after which changes occurred in the organization and availability of health services. Therefore, the findings primarily reflect the state of the health system in the pre-pandemic period, and our future research should examine whether the patterns and determinants of unmet health needs of older adults have changed over time.

## 5 Conclusion

National health surveys enable the detection of problems in the health system, the analysis of health care quality indicators, but also predictors of unfulfilled health needs. Reforms of the healthcare system in Serbia have so far been focused on improving infrastructure and technology and implementing an integrated healthcare information system. Information about the factors that prevent the provision of health care services is important in adapting health resources to the needs of the population. All indicators can be used in order to improve the quality of health services. The results of the survey show that in order to design more accessible health care models, a transparent and comprehensive analysis of the economic situation of the older adult and its impact on the possibility of obtaining the necessary health care is needed.

## Data Availability

The raw data supporting the conclusions of this article will be made available by the authors, without undue reservation.
